# Abnormal expressions of immune response-related genes in RA bone marrow cells

**DOI:** 10.1186/ar3640

**Published:** 2012-02-09

**Authors:** Hooi-Ming Lee, Chieko Aoki, Yasunori Shimaoka, Kensuke Ochi, Takahiro Ochi, Norihiro Nishimoto

**Affiliations:** 1 Graduate School of Frontier Biosciences, Osaka University, 1-3 Yamada-Oka, Suita, Osaka 565-0871, Japan; 2 Laboratory of Immune Regulation, Wakayama Medical University, 105 Saito Bio Innovation Center, 7-7-20 Saito-Asagi, Ibaraki, Osaka 567-0085, Japan; 3 Yukioka Hospital, 2-2-3 Ukita, Kita-ku, Osaka 530-0021, Japan; 4 Kawasaki Municipal Kawasaki Hospital, 12-1 Shinkawa-dori, Kawasaki-ku, Kawasaki, Kanagawa 210-0013, Japan; 5 Osaka Police Hospital, 10-31 Kitayama-chou, Tennoji-ku, Osaka 543-0035, Japan

## Background

Rheumatoid arthritis (RA) is a systemic autoimmune disease characterized by chronic synovitis that progresses to destruction of cartilage and bone. Bone marrow (BM) cells have been shown to contribute to this pathogenesis. In this study, we compared differentially expressed molecules in BM cells from RA and osteoarthritis (OA) patients and analyzed abnormal regulatory networks to identify the role of BM cells in RA.

## Materials and methods

Gene expression profiles (GEPs) in BM-derived mononuclear cells from 9 RA and 10 OA patients were obtained by DNA microarray. Up- and down-regulated genes were identified by comparing the GEPs from the two patient groups. Bioinformatics was performed by Expression Analysis Systemic Explorer (EASE) 2.0 based on gene ontology, followed by network pathway analysis with Ingenuity Pathways Analysis (IPA) 7.5.

## Results

The BM mononuclear cells showed 764 up-regulated and 1,910 down-regulated genes in RA patients relative to the OA group. EASE revealed that the gene category response to external stimulus, which included the gene category immune response, was overrepresented by the up-regulated genes. So too were the gene categories signal transduction and phosphate metabolism. Down-regulated genes were dominantly classified in three gene categories: cell proliferation, which included mitotic cell cycle, DNA replication and chromosome cycle, and DNA metabolism. Most genes in these categories overlapped with each other. IPA analysis showed that the up-regulated genes in immune response were highly relevant to the antigen presentation pathway and to interferon signaling. The major histocompatibility complex (MHC) class I molecules, HLA-E, HLA-F, and HLA-G, tapasin (TAP) and TAP binding protein, both of which are involved in peptide antigen binding and presentation via MHC class I molecules, are depicted in the immune response molecule networks. Interferon gamma and interleukin 8 were overexpressed and found to play central roles in these networks.

## Conclusions

Abnormal regulatory networks in the immune response and cell cycle categories were identified in BM mononuclear cells from RA patients, indicating that the BM is pathologically involved in RA.

